# Comparison of ox-LDL Levels in Diabetic Patients with Normo-, Micro-, and Macroalbuminuria with Their First Degree Relatives and the Healthy Control Group

**DOI:** 10.1155/2012/167154

**Published:** 2012-11-01

**Authors:** Parisa Behzadi, Firouzeh Torabi, Massoud Amini, Ashraf Aminorroaya

**Affiliations:** ^1^Isfahan Endocrine and Metabolism Research Center, Isfahan University of Medical Sciences, Isfahan, Iran; ^2^Internal Medicine and Endocrinology, Isfahan Endocrine and Metabolism Research Center, Isfahan University of Medical Sciences, Sedigheh Tahereh Research Complex, Khorram Street, 8187698191 Isfahan, Iran

## Abstract

Oxidized low density lipoprotein (ox-LDL) is a product of oxidative stress. In this cross-sectional study, we compared the ox-LDL concentrations in diabetic patients with normoalbuminuria (*n* = 28), microalbuminuria (*n* = 28), and macroalbuminuria (*n* = 28) with their first degree relatives (*n* = 28) and healthy control people (*n* = 31). They were selected by consecutive patient selection method. The ox-LDL level was assayed using ELISA. We measured blood pressure, lipid profile, fasting plasma glucose (FPG), and HbA1c in all groups. There was no significant difference in ox-LDL concentrations among normoalbuminuric, microalbuminuric, and macroalbuminuric diabetic groups. In diabetic patients with micro- and macroalbuminuria, ox-LDL concentration was higher than their first degree relatives (*P* = 0.04 and *P* = 0.03) and control group (*P* = 0.001 and *P* = 0.03, resp.). In normoalbuminuric diabetic persons, ox-LDL concentration was just higher than that of healthy people (*P* = 0.02). There was no statistically significant difference in ox-LDL levels between normoalbuminuric diabetic patients and their first degree relatives. In conclusion, the presence and progression of albuminuria in diabetic patients are not related to ox-LDL concentration and genetic predisposition influences the plasma OX-LDL level. Larger sample size is needed to confirm this conclusion in future studies.

## 1. Introduction

Diabetes mellitus is a common metabolic disease and has been reported as a state of oxidative stress, inflammation, and endothelial dysfunction [1]. One of the ominous complications of this disease is diabetic nephropathy which is a major cause of end stage renal disease worldwide. There are some evidences that oxidative stress is a main culprit of diabetic nephropathy, and increased levels of oxidized low density lipoprotein (ox-LDL) immune complexes were reported in patients with diabetic nephropathy [2]. The oxidative change of LDL affects its clearance and cause cytotoxic and immunogenic effects. We also know that ox-LDL immune-complexes contribute to the development of atherosclerosis [3–5]. However, the results of several researches suggest that ox-LDL contributes to the initiation and progression of diabetic nephropathy. If we consider diabetic nephropathy as sequences of the events, it is starting with endothelial cell dysfunction which is promoted by ox-LDL [6, 7]. Some mechanisms have been expressed to elucidate the effects of ox-LDL on glomerulus. Evidences have been reported that the chemokine cxcl16 is the main receptor in podocyte mediating the uptake of ox-LDL and therefore, initiate a pathologic biochemical pathway which affects protein trafficking and podocyte survival [8]. 

Findings of another research in USA demonstrated that ox-LDL immune complexes stimulated collagen IV production in mesangial cells and therefore exert progressive mesangial thickening in diabetic nephropathy [9]. We have some data supporting that ox-LDL immune complexes (ox-LDL-ICs) accelerate atherosclerosis in diabetic patients and glomeruosclerosis which occurs in diabetic nephropathy is analogous to that of atherosclerosis and is relevant to mesangial cell production of collagen IV. In vitro experiments have shown that ox-LDL-ICs stimulate human mesangial cells to produce collagen IV, but a minority of biopsy specimens of patients with diabetic nephropathy (2 of 16) contained ox-LDL-ICs which existed in the sclerotic rather than mesangial regions. So it is difficult to see a definite relation between ox-LDL-ICs and human diabetic nephropathy [10]. With this background, we decided to investigate the ox-LDL levels in diabetic patients with normo-, micro-, and macroalbuminuria in comparison with that of their first degree relatives and the healthy control group. 

## 2. Methods

In this cross-sectional study, we investigated three groups of type 2 diabetic patients with normo-, micro-, and macroalbuminuria (28 patients in each group). They were selected by consecutive patient selection method from people coming to the Isfahan Endocrine and Metabolism Research Center (IEMRC) of Isfahan University of Medical Sciences. Individuals with kidney transplantation were excluded from the study [11].

We measured albumin and creatinine in a morning sample of urine by the kit obtained from Pars Azmoon Company using BT3000, Zinsser Analytic, Germany. Albumin creatinine ration (ACR) <30 mg/g was considered as normoalbuminuria, 30 mg/g ≤ ACR ≤ 300 mg/g as microalbuminuria, and ACR > 300 mg/g as macroalbuminuria [12].

The first degree relatives of diabetic patients were recruited by telephone call. After 8 hours of overnight fasting, glucose tolerance test (GTT) was performed and people with normal glucose tolerance were selected for the study [13]. The control healthy group were chosen from people coming to the Blood Bank of Isfahan city. Those who had fasting plasma glucose less than 100 mg/dL were enrolled the study [14]. We measured blood pressure at the morning in the supine position and before sample taking. We asked and recorded duration of diabetes in all the diabetic patients. We measured ox-LDL concentration using ELISA kit, DRG Company, Germany, by model Anthos 2010, Zinsser Analytic, Germany [15]. The technique of measuring the plasma ox-LDL level is by a competitive ELISA utilizing a specific murine monoclonal antibody, mAb-4E6, which Holvoet used in his assays for the first time [16]. However, the assay kit is a capture ELISA (sandwich ELISA) in which the wells of the microtiter plates are coated with the capture antibody mAb-4E6. OX-LDL ELISA is a solid-phase two-site enzyme immunoassay. It is based on the direct sandwich technique in which two monoclonal antibodies are directed against separate antigenic determinant on the oxidized apolipoprotein B molecules. During incubation, OX-LDL in the sample reacts with anti-ox-LDL antibodies bound to the microtitration well. After washing that removes nonreactive plasma component, a peroxidase conjugated anti-apolipoprotein B antibody recognizes the ox-LDL bound to the solid phase. After a second incubation and a simple washing step that removes unbound enzyme-labeled antibody, the bound conjugate is detected by reaction with tetramethylbenzidine (TMB). The reaction is stopped by adding acid to give a colorimetric end point that is read spectrophotometrically at 450 nm.

Total cholesterol was measured by CHOD-POP and high density lipoprotein by HDL-C enzymatic methods. Triglyceride and FPG were measured by GPO-PAP and GOD-PAP enzymatic methods, respectively. LDL cholesterol concentration was calculated by the Friedewaled formula. HbA1c concentration was measured by ion exchange chromatography.

Data were expressed as mean and standard deviation (SD) and were analyzed using SPSS software version 13. Age and gender and duration of diabetes entered the analysis as covariates using univariate analysis. *P* values less than 0.05 were statistically considered significant. Regression test was performed in order to investigate the correlation between ox-LDL values and other variables such as lipoproteins, HbA1c, FPG, and blood pressure.

## 3. Results

**Figure 1:**
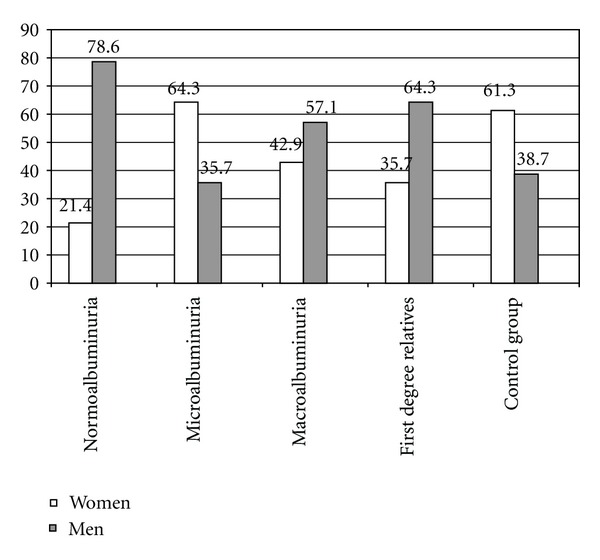
Frequency distribution of sex in the five groups of the study.

The frequency distribution of sex is shown in Figure 1. Males are maximum in the normoalbuminurics and minimum in the microalbuminuric group. Results of comparing age, duration of diabetes, and systolic and diastolic blood pressure are shown in Table 1. The first degree relatives of diabetic patients are significantly younger than the control group and the diabetic groups. The diabetic patients with macroalbuminuria are significantly older than all other groups (Table 1). Systolic but not diastolic blood pressure was higher in macroalbuminuric compared with normoalbuminuric (*P* = 0.0001), microalbuminuric diabetic patients (*P* = 0.003), the first degree relatives (*P* = 0.0001), and the control group (*P* = 0.0001).

The concentrations of ox-LDL have been expressed as mean (SD) in Table 2. There is no statistically significant difference in ox-LDL levels between the normoalbuminuric patients and the first degree relatives of diabetic patients (*P* = 0.54). However, in this group the mean of ox-LDL was higher than the control group (*P* = 0.02). In diabetic patients with micro- and macroalbuminuria, the mean of OX-LDL concentration is significantly higher than that of their first degree relatives (*P* = 0.04, *P* = 0.03) and the control group (*P* = 0.001, *P* = 0.0001). OX-LDL concentration was not significantly higher in macroalbuminuric patients than microalbuminuric and normoabluminuric diabetic patients. There is no significant difference between concentrations of OX-LDL between microalbuminuric and normoabluminuric groups, too. 

There is no statistically significant difference in ox-LDL concentration between females and males in all groups of diabetic patients, their first degree relatives, and healthy people (*P* = NS). Age, sex, and duration of diabetes had no significant effect on ox-LDL concentrations as far as we entered these variables as covariates in univariate analysis.

Table 3 shows the mean concentrations of lipids, FPG, and HbA1c in five groups. Total cholesterol levels in all the 3 diabetic groups were higher than the first degree relatives (*P* = 0.0001, *P* = 0.002 and *P* = 0.02). The macroalbumiuric patients had higher levels of total cholesterol in comparison with the control group (*P* = 0.035). The LDL-C concentrations were higher in normo-, micro-, and macroalbuminuric diabetic patients than the control group (*P* = 0.04, *P* = 0.02, and *P* = 0.03). There was no statistically significant difference in HDL-C concentrations among the 5 groups. Triglyceride levels were higher in normo-, micro-, and macroalbuminuric diabetic groups than the first degree relatives (*P* = 0.001, *P* = 0.021, and *P* = 0.04) and the control group (*P* = 0.0001, *P* = 0.01, and *P* = 0.011). Therefore, normo-, micro-, and macroalbuminuric diabetic patients had similar lipid profile. FPG was not significantly different between micro- and macroalbuminuric diabetic patients (*P* = 0.42). As we expected, HbA1c was significantly higher in micro- and macroalbuminuric diabetic patients than all other groups (*P* = 0.0001). Diabetic patients with macro- and microalbuminuria had higher fasting plasma glucose than normoalbuminuric diabetic patients (*P* = 0.032). However, there were no statistically significant difference between FPG and HbA1c concentrations between micro- and macroalbuminuric diabetic patients (*P* = NS). 

Table 4 shows the correlation coefficients comparing the levels of ox-LDL with other variables. There is a weak inverse correlation between plasma ox-LDL and fasting plasma glucose only in the control group (*r* = −0.37, *P* = 0.05). There is a direct correlation between plasma ox-LDL and total cholesterol in the same group (*r* = 0.49, *P* = 0.001).

There was a correlation between ox-LDL and HbA1c levels just in microabuminuric diabetic group but not in the other four groups (*r* = −0.38, *P* = 0.001). 

## 4. Discussion

In this study, we compared ox-LDL level in the three groups of diabetic patients including people with normal albumin excretion in urine, microalbuminuria, and macroalbuminuria. We investigated ox-LDL concentration in two nondiabetic groups including the first degree relatives of the above-mentioned diabetic patients and the healthy control group with no apparent genetic relationship. 

We found that ox-LDL level is significantly higher in all the three diabetic groups than the control group, which confirms the presence of oxidative stress in diabetes.

In addition, our findings showed that ox-LDL concentration is significantly higher in macro- and microalbuminuric patients than the first degree relatives of diabetic patients. 

However, there is no significant difference in ox-LDL level among normo-, micro-, and macroabuminuric diabetic patients (Table 2). It proposes that the presence and progression of proteinuria in diabetic patients is not dependent on the ox-LDL concentration. 

There was no significant difference in ox-LDL level between the normoalbuminuric diabetic patients and the first degree relatives group. It seems that both genetic predisposition and oxidative stress in diabetes may affect LDL oxidation. 

However, a possible cross-reactivity of the antibody with nonoxidized LDL and interference of immune complexes formed by ox-LDL and the corresponding auto antibodies should be considered in the interpretation of our findings. 

Other studies have been done in this field. The work of Nakhjavani et al. demonstrated the association of macroalbuminuria with TGF-beta and ox-LDL in type 2 diabetic patients. They selected two groups of diabetic patients, consisting of patients with macroalbuminuria and those with normal albumin excretion. Their results are contradictory to our results; that is, ox-LDL concentrations were significantly higher in macroalbuminuric patients in comparison with normoalbuminurics. Besides, their study confirms the role of TGF-beta along with ox-LDL in the development of diabetic nephropathy. They also found a significant correlation between albumin excretion rate and ox-LDL concentration in macroalbuminuric patients [17].

Atchley et al. in medical university of South Carolina showed that high concentrations of ox-LDL immune complexes are associated with abnormal proteinuria [18]. 

A research in Japan showed that ox-LDL level is significantly increased in macroalbuminuric diabetic patients in comparison with micro- and normoabluminuric persons and the healthy control group. In contrast to our findings, those studies considered the ox-LDL as an effective factor in the development of diabetic nephropathy [11].

In a study in Italy, microalbuminuric patients had elevated levels of ox-LDL compared with the normoalbuminuric and the control healthy group [19]. 

Findings of a recently published cohort study by Lopes-Virella et al. demonstrated that during 14–20 years followup of 302 type 1 diabetic patients, high levels of ox-LDL in circulating immune complexes were associated with increased odds of developing abnormal proteinuria [20]. The common feature of the mentioned studies is the association of ox-LDL with proteinuria in diabetic nephropathy. However, we did not come to the same results. The novel aspect of our study is having the first degree relatives of diabetic patients as a second control group and demonstration of genetic predisposition effect on ox-LDL level. However, the groups of patients and controls are of small size. 

There is a study by Rashidi et al. which has a new different feature. They paid attention to renal function as a confounding factor and showed that when the effect of renal function was controlled for, there was not a significant correlation between proteinuria and high ox-LDL concentration in type 2 diabetic nephropathy. But other oxidant markers such as malondialdehyde (MDA) and extracellular superoxide dismutase (EC-SOD) have significant association with proteinuria, independent of renal function [21]. However, in spite of evidences that demonstrate toxic effect of ox-LDL and its immune complexes on renal cells, the causative role of ox-LDL in diabetic nephropathy has not been established and final conclusion needs more evidences.

## 5. Conclusion

The presence and progression of albuminuria in diabetic patients are not related to ox-LDL concentration, and genetic predisposition influences the plasma level. Larger sample size is needed to confirm this conclusion in future studies.

## Figures and Tables

**Table 1:** Comparisons of age and systolic and diastolic blood pressure in individuals of five groups and duration of diabetes in three groups of diabetic patients.

	Number	Age (year)	Duration of diabetes	Systolic blood pressure	Diastolic blood pressure
Normoalbuminuric diabetics	28	44.6 ± 8.1	3.9 ± 3.6	118 ± 14	68 ± 12
Microalbuminuric diabetics	28	49.1 ± 10	7.7 ± 4	122 ± 14	75 ± 14
Macroalbuminuric diabetics	28	57.4 ± 8.8	16.9 ± 4.1	136 ± 19	79 ± 6
First degree relatives	28	26.3 ± 11		105 ± 12	69 ± 6
Control group	31	44.5 ± 7.3		110 ±10	69 ± 6

**Table 2:** Mean and standard deviation of ox-LDL concentration in the five groups of the study.

Groups	Mean ± SD	CI 95%
Normoabluminuric diabetic patients	14.2 ± 2.7	13.1–15.2
Microabuminuric diabetics	15.08 ± 3.9	13.5–16.6
Macroalbuminuric diabetics	15.47 ± 4.7	13.6–17.3
First degree relatives of diabetic patients	12.3 ± 6.3	9.8–14.8
Control group	10.7 ± 2.2	9.9 ± 11.53

**Table 3:** Comparison of lipid parameters, fasting plasma glucose, and HbA1c in all groups of the study.

Groups	Total cholesterol	LDL-C	HDL-C	FBG	HbA1c
	mg/dL	mg/dL	mg/dL	mg/dL	%
Normoalbuminuric diabetic patients	198 ± 33	127 ± 35	51 ± 56	171 ± 58	7.8 ± 1.5
Microalbuminuric diabetic patients	207 ± 60	127 ± 32	42 ± 19	199 ± 18	8.6 ± 2.3
Macroalbuminuric diabetic patients	217 ± 81	120 ± 31	42 ± 10	207 ± 68	10 ± 3
First degree relatives	154 ± 39	78 ± 20	50 ± 11	81 ± 10	5.6 ± 0.4
Control group	178 ± 26	87 ± 22	37 ± 10	81 ± 17	5 ± 0.5

^∗^Abbreviations: HDL-C: high density lipoprotein cholesterol, LDL-C: low density lipoprotein cholesterol, FBG: fasting blood glucose, HbA1: glycosylated hemoglobin.

**Table 4:** Spearman's correlation coefficients between lipid parameters, FBG, HbA1c, and OX-LDL in all groups of the study.

Groups	Total cholesterol	LDL-C	HDL-C	TG	HbA1c	FBG
	*r * Pearson	*r * Pearson	*r * Pearson	*r * Pearson	*r * Pearson	*r * Pearson
Ox-LDL of normoalbuminurics	−0.07	−0.085	−0.25	−0.21	−0.024	−0.220
Ox-LDL of microalbuminurics	−0.086	−0.2	−0.28	0.28	−0.379	0.36
Ox-LDL of macroalbuminurics	−0.148	0.066	0.001	−0.139	−0.136	−0.129
Ox-LDL of first degree relatives	−0.26	0.21	−0.029	0.26	0.03	−0.093
Ox-LDL of control group	0.49	0.33	0.092	0.05	0.057	−0.37

^∗^Abbreviations: HDL: high density lipoprotein cholesterol, LDL: low density lipoprotein cholesterol, FBG: fasting blood glucose, HbA: glycosylated hemoglobin.
